# Anti-reflux procedure for difficult-to-treat asthmatic children, case report and literature review

**DOI:** 10.1186/2049-6958-7-28

**Published:** 2012-09-14

**Authors:** Zhi-Wei Hu, Zhong-Gao Wang, Ji-Min Wu, Song-Tao Tan

**Affiliations:** 1 Xuanwu Hospital of Capital Medical University, Beijing, China; 2 Center for GERD, Second Artillery General Hospital of Beijing Normal University, Beijing, China; 3 Center for GERD, Wu Jing Zong Dui Hospital of Guangdong Province, Guangzhou, China; 4 No. 16 Xinwai Street, Xicheng district, Beijing 100088, China

**Keywords:** Gastroesophageal reflux disease, Difficult-to-treat Asthma, Children, Stretta frequency, Laparoscopic fundoplication

## Abstract

Gastroesophageal reflux disease (GERD) is a commonly encountered condition in children, which at times causes respiratory distress, such as asthmatic symptoms, and results in serious morbidity and even mortality. The complexity is sometimes so obscure, that it can cause paradoxical diagnoses and treatment. Here we present two cases of children with difficult-to-treat asthmatic symptoms, which were eventually found to be related to GERD. The two children were treated with anti-reflux procedures and both became symptom free. Literature was also reviewed to shed a light into this complex disease.

## Case I

It was a 12 year-old girl, who had frequent cough and wheezing which required hospital visits and intravenous anti-asthmatic medication every year, since she was two years old. However, after she was ten her symptoms worsened when her wheezing became daily episodes accompanied with violent cough which could last for one or two hours before remission, making her difficult to lie down at midnight. She also reported sneezing, eye-itching and tearing before the onset of cough and wheezing, but no notable heartburn or regurgitation was recalled. During the years, despite her longing to improve grades in school, she often had to cut school due to hospital visits or fatigue, Her family had consulted five major hospitals in different provinces, in addition to almost all the local hospitals. The diagnosis was always the same: allergic asthma. Her spirometry test showed 16% improvement in FEV_1_ after inhaling albuterol and the only one positive skin prick test result was house-dust mites four years ago. Despite maximum dose of oral corticosteroid and Beta-agonists inhaling, or traditional Chinese medicine, her symptoms seemed to be uncontrollable. At last, she came to us for gastroesophageal reflux (GER) evaluation to find out if the asthmatic symptom is second to GER. The routine 24-hour pH monitoring showed pathological acid reflux (DeMeester score: 25.45), which was more severe in supine position. And the longest reflux (18.1 min) occurred at one midnight when she had an asthma attack. According to the data we concluded that her asthma was GER related. We conducted Stretta Frequency (SRF) procedure on the patient after having approval from the hospital’s ethics research committee and obtaining parental consent from the patient’s family. Since the day of the anti-reflux treatment, the patient can sleep well and until now her handicapping symptoms have disappeared for 31 months without medication.

## Case 2

In this case the patient is a boy. Since he was born in 1997, his mother found he was difficult to feed due to frequent belching and regurgitation of milk into his mouth and nasal cavity. At the age of six, wheezing, short of breath and productive chough caused him to be hospitalized and the disease was treated as “pneumonia”. Since then until he aged ten, his repeated “pneumonia” forced him to be taken into hospital almost every month, with each hospital stay lasting about ten days. Through the years he usually had productive cough for one or two days before onset of wheezing and short of breath. He was also diagnosed as affected with “allergic asthma” in other hospitals though allergen was not identified. All kinds of anti-asthmatic medications (oral, intravenous or inhaled) had been tried, which were all helpless in preventing the disease from getting worse. Despite constant and marked symptoms of belching and regurgitation, accompanied by astigmatism and sore throat started since age ten, these symptoms were neglected and untreated by his family and physicians. Through the Internet, the desperate mother found us and brought the boy for GER evaluation.

**Figure 1:**
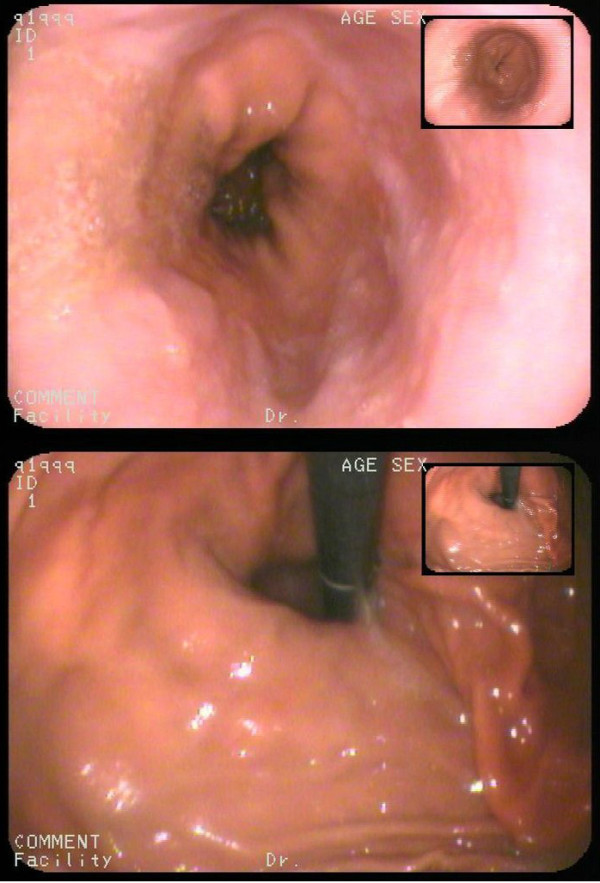
**In case 2, a hiatus hernia without esophagitis was identified under endoscopy, which was considered as the cause of the boy’s persistent and evident GER and then the difficult-to-treat asthmatic symptom.** And this anatomical defect indicates a surgical intervention.

On admission, the patient was a lean adolescent appearing to be healthy with no signs of respiratory distress. His chest film showed mild emphysema, total IgE plasma level was normal (12.90 IU/ml) and the esophagogastroduodenoscopy showed a sliding hiatal hernia and non-erosive reflux disease (NERD) (Figure 1). A following esophagus manometry also found the hernia and showed a hypotensive short lower esophageal sphincter (LES) and abnormal esophageal contraction waves. We concluded that his hiatal hernia, GER and asthmatic symptoms were related. Laparoscopic Nissen fundoplication (LNF) and hiatal hernia repair were performed on him. After the surgery, we have followed up the boy for 15 months and found that episodic respiratory distress , “pneumonia”, and his belching and regurgitation were cleared up.

## Discussion

Gastroesophageal reflux disease (GERD) is a commonly encountered condition in childhood [1]. Reflux-associated extraesophageal symptoms in infants and children include serious events, such as oxygen desaturation, episodes of apnea, recurrent aspiration, as well as symptoms such as asthma, bronchitis, irritability, and sleep disturbances, which apparently may result in serious morbidity, even mortality [2,3]. In the first three months of life, postprandial reflux is considered a physiological event which gradually decreases and disappears by one year of age [4]. The progressive decrease in episodes is due to maturation of the LES and to acquisition of sitting and standing. However, some children have persistent regurgitation or reflux after the first year of age. Their reflux is not only associated with feeding but also with backwardness, behavioral disorders such as irritability, unjustified crying and sudden waking, and persistent esophageal hiatus defect [5].

Similar to GERD, asthma is also a common disease with chronic complex inflammatory airway disorder, which is characterized by variable degrees of recurring symptoms of airflow obstruction and bronchial hyper responsiveness [6]. Although the majority of asthma patients may obtain the targeted level of control, some patients will not achieve control even with the best therapy [7]. Patients who do not reach an acceptable level of control with reliever medication plus two or more controllers can be considered to have difficult-to-treat asthma [8]. The lives of children with difficult-to-treat asthma are severely disrupted with frequent hospital visits, school absence, and limitation of normal activities. Behavioural problems and a lower quality of life are more pronounced in those children [9]. Persistent airflow limitation is present in a proportion of these patients [10]. Although they probably account for less than 5% of all children with asthma, the management of this group of children is difficult, with little evidence to guide the choice of further treatment for those who remain symptomatic even after the use of regular systemic corticosteroids.

The association between asthma and GER has been debated for decades after Sir William Osler first observed the association between worsening asthma and distended stomach in 1892 [11]. The prevalence of GERD in children with asthma ranged from 19.3% to 80.0% and averaged 22.8%. In patients with asthma, the average prevalence of abnormal esophageal pH was 68.2% and of esophagitis was 35.6%. GERD was found in about 49% of patients with childhood difficult-to-treat asthma [12] and a hiatal hernia often predicts a higher risk of GERD due to the anatomical defect [13].

The diagnosis of GER is not easy in some children with asthmatic symptom. GER may be present with bronchiolitis, pneumonitis, and even failure to thrive. Other common GER respiratory manifestations are chronic coughing, asthma, laryngeal spasm, apnea, stridor, pulmonary dysplasia, and cyanotic crises. Nocturnal wheezing or coughing with inadequate response to medical treatment for asthma, negative family history of atopy, and early onset of bronchial hyperreactivity can distinguish these patients [14]. GER typically has symptoms such as heartburn and/or regurgitation. However the prevalence of asymptomatic acid reflux in patients with asthma varies between 10% and 62% according to the underlying severity of the asthma and the measure used to identify symptoms [15]. Thus, there is a need for tests to predict the presence of GER among children with difficult-to-treat asthma. 24-h intra-esophageal pH-monitoring is one of the current reference-standard methods for GER assessment in children [16]. Multichannel intraluminal impedance and pH (MII-pH) monitoring can detect anterograde or retrograde acid or non-acid bolus and determine the composition (liquid, gas and mixed) movement into the esophagus, as well as the height reached by the refluxate [17]. Endoscopy is sensitive for esophagitis and hiatal hernia. Esophagus manometry is valuable for demonstration of esophageal dyskinesia, hypotonia of the LES or the presence of hiatal hernia. An easy-to-conduct barium swallow study is useful to detect hiatus hernia or spontaneous reflux. These modalities may have important diagnostic and therapeutic implications for children with reflux-related respiratory problems. Recently, a study by Dal Negro et al. showed that esophageal acidification has a good level of both sensitivity and specificity by enhancing the MCh response in FEV1 only in the presence of acid GER. This test could be a potentially useful tool for better selection of GER-related asthma from the asthmatics in clinical practice [18].

Reflux management strategies focus on two main areas: First, to use effective agents to reduce acid secretion and thus the likely damage to the esophagus and lungs from their long-term or repeated exposure to acidic gastric contents. Proton pump inhibitor (PPI) therapy is currently the most accepted medical therapy in children, as it is in adults. However, PPI failed to show benefit in terms of asthma control in children with GERD in most of the well-designed studies [19]. Despite the current lack of population based studies and longitudinal studies, PPI still could be a valuable empiric treatment for well selected asthmatic children with GERD. Second, to correct some of the anatomic abnormalities. By artificial means, it is to increase tonicity and to restore functions of the lower esophageal sphincter to some degree. For a patient who has difficult-to-treat asthma, whether the etiology is primary or secondary to an underling disorder such as GERD must be clarified. To treat cases of GER related asthmatic symptoms, fundoplication or some other procedures should be considered for patients who have failed maximal medical therapy, who are not proper candidates to undergo medical therapy for some special reasons, and who have hiatal hernia contributing to the reflux. It is essential to confirm that any existing anatomic or neurophysiologic defects are either remedied first or are considered as one part of the GERD management as well. Since 2006 when we established a Center for GERD to diagnose and to treat GERD patients with asthmatic symptoms using SRF or LNF, some good results have been documented for adult patients [20]. Effectiveness of surgical therapy in different uncontrolled series in children with severe persistent asthmas have also been reported [21-23], which shed a light on potential alternative or promising therapy for difficult-to-treat or uncontrolled asthmatic for children.

## Consent

Written informed consents were obtained from the patients for publication of this case report and for any associated images. A copy of the written consents is available for review by the Editor-in-Chief of this journal.

## Abbreviations

GERD: Gastroesophageal Reflux Disease; SRF: Stretta frequency; LES: Lower esophageal sphincter.

## Competing interests

The authors declare that they have no competing interests.

## Authors’ contributions

ZW Hu studied and analyzed the two cases, conducted literature reviews, and drafted the manuscript. ZG Wang designed the study and helped to draft the manuscript. JM Wu and ST Tan carried out the study, collected the data and helped to draft the manuscript. All authors read and approved the final manuscript.

## Authors’ information

ZW Hu, the resident in training program in Center for GERD of Second Artillery General Hospital; ZG Wang, the professor and director of Center for GERD of Second Artillery General Hospital, Professor and Director of vascular institution of Xuanwu Hospital of Capital Medical University Capital, Life Long President of China Vascular Society, Vice President of International Society of Vascular Surgery and Academician of China Academy of Science. JM Wu, director of Center for GERD of Second Artillery General Hospital, ST Tan, director of Center for GERD, Wu Jing Zong Dui Hospital of Guangdong Province.
